# Integrated interdepartmental management of mandibular fractures involving third molars

**DOI:** 10.6026/973206300212931

**Published:** 2025-08-31

**Authors:** Neha Jain, Ashutoshdutt Pathak, Shahid Khalid Ponneth, Rahul Sharma, Sabhrant Singh, Nitin Kumar Mishra

**Affiliations:** 1 Department of Oral and Maxillofacial Surgery, People Dental Academy, Bhopal, Madhya Pradesh, India; 2 Department of Oral and Maxillofacial Surgery, People College of Dental Sciences and Research Center, Bhopal, Madhya Pradesh, India; 3 Department of Dentistry, Hamad Medical Corporation, Qatar; 4 Department of Dentistry, District Hospital, Anuppur, Madhya Pradesh, India

**Keywords:** Mandibular fracture, pathological fracture, open reduction internal fixation (ORIF), prosthodontic rehabilitation, odontogenic cyst, multidisciplinary management, bone healing, maxillofacial surgery

## Abstract

Over the course of a year, this study assessed the combined surgical and prosthodontic treatment of 30 patients' pathological mandibular fractures linked to third molars. Open reduction and internal fixation were used to treat patients with benign pathologies that compromised bone integrity. After the initial healing period, prosthodontic rehabilitation was performed. With few complications, the results showed a high rate of bone healing, functional restoration and patient satisfaction. Data demonstrate how well a coordinated, multidisciplinary approach can restore the structural and functional integrity of these intricate mandibular fractures.

## Background:

Because of the mandible's anatomical complexity and functional significance, mandibular fractures are among the most frequent facial injuries seen in maxillofacial trauma and pose serious clinical challenges. From simple linear fractures to comminuted or pathological variants that require complex treatment protocols, these fractures can be caused by a variety of aetiologies, such as falls, physical assaults, traffic accidents and sports injuries [1, 2]. A significant percentage of maxillofacial fractures in both civilian and military populations are caused by trauma to the mandible, which is especially vulnerable due to its prominent position and mobility [3]. Over time, mandibular fracture management has changed dramatically, with improvements in imaging, fixation methods and biomaterials leading to better results. To avoid complications like malocclusion, infection, non-union and functional impairment, prompt intervention and accurate diagnosis are essential [4]. Depending on the location and severity of the fracture as well as the patient's overall health, treatment options range from closed reduction and maxillomandibular fixation (MMF) to open reduction and internal fixation (ORIF) [5]. Reconstruction techniques utilising bone grafts or free vascularised flaps may be required in complex cases, such as those involving segmental mandibular loss brought on by trauma or disease, in order to restore both form and function [6, 7]. It has been demonstrated that using allogeneic bone grafts or specially designed reconstruction plates can restore both structural integrity and aesthetics in cases of large mandibular defects, highlighting the need for customised treatment regimens [1, 6]. Since it aids in the restoration of speech, facial harmony and mastication, post-operative prosthetic rehabilitation is a crucial stage in the overall management of mandibular fractures [8]. Furthermore, patients may be at risk for fractures or have their healing trajectory impacted by underlying systemic conditions like osteoporosis, underscoring the significance of a multidisciplinary approach in management [9]. Therefore in order to improve treatment protocols and stop recurrence, epidemiological studies have emphasised the importance of careful documentation and analysis of fracture patterns [10]. Significant patterns in incidence, contributing factors and treatment-related complications were found in a ten-year study of mandibular fractures, offering important new information for clinical practice [11]. Therefore, it is of interest to show the importance of accurate diagnosis, evidence-based treatment planning and comprehensive patient care.

## Methodology:

The integration of prosthodontics and oral surgery in the treatment of pathological mandibular fractures linked to third molars was the focus of this one-year prospective interventional study. Thirty patients between the ages of 18 and 65 were recruited; all of them had pathological mandibular fractures involving or close to impacted or erupted third molars that were confirmed by radiography and clinical examination. Patients with benign pathological conditions that required surgical management, such as cysts or odontogenic tumours, were included in the inclusion criteria. Malignant pathology, previous radiation therapy to the jaws, uncontrolled systemic illnesses, poor oral hygiene and noncompliance with follow-up were among the exclusion criteria. Preoperative evaluations included a thorough clinical examination, radiographic imaging using cone-beam computed tomography (CBCT) and panoramic radiographs and baseline assessments of functional status and patient-reported outcomes, such as pain scores and quality of life related to oral health (OHIP-14). In the oral surgery phase, the involved third molar was extracted (if indicated), the pathological tissue was debrided and the fracture was openly reduced with internal fixation (ORIF) using titanium plates and screws, either with or without bone grafting, depending on the degree of bone loss. The patient was given postoperative antibiotics, analgesics and dietary recommendations after occlusion were confirmed intraoperatively. When required, initial stabilisation was applied using occlusal splints or guiding elastics. The prosthodontic rehabilitation phase started after the initial healing was confirmed by radiography and clinical examination at around 6 to 8 weeks. The creation of final restorations, such as crowns, detachable partial dentures, or occlusal splints, that were designed to restore function, aesthetics and occlusal balance, was part of this phase. Restoring masticatory efficiency and distributing functional loads uniformly throughout the healed fracture site were priorities. Clinical exams, radiographic evaluations and functional assessments were performed at each follow-up visit, which occurred at regular intervals of one week, two weeks, six weeks, three months, six months and one year. Clinical evidence of bone healing, lack of mobility at the fracture site, occlusal stability, function restoration, patient satisfaction and complication rates like infection, non-union, or prosthetic failure were among the outcome measures. The significance level was set at p < 0.05 and the data were examined using the proper statistical techniques. Prior to inclusion, all participants provided written, informed consent and the institutional review board granted ethical clearance.

## Results:

The study included 30 patients, with a mean age of 36.7 years (range 19-63 years), 12 of who were female and 18 of who were male. The third molar region was affected by unilateral pathological mandibular fractures in all of the patients. Benign odontogenic tumours (26.7%), chronic pericoronitis with associated bone resorption (20%) and odontogenic cysts (53.3%) were the most prevalent underlying pathology. In every instance, open reduction and internal fixation (ORIF) surgery was successfully completed, with the third molar extracted when necessary. Ten patients (33.3%) required bone grafting because of severe bone loss. 27 patients (90%) experienced uneventful healing, whereas 3 patients (10%) experienced minor complications like delayed wound healing (1 case) and localised infection (2 cases). Following an average healing period of 7.5 weeks, prosthodontic rehabilitation was started. Ten of the thirty patients underwent rehabilitation with removable partial dentures, while the other twenty received fixed prostheses. At three and six months after surgery, clinical and radiographic evaluations showed that 28 patients (93.3%) had stable occlusion, satisfactory prosthetic function and no pathology recurrence. Occlusal discrepancies necessitated minor prosthetic adjustments for two patients. In the final follow-up, 90% of participants reported satisfactory functional and aesthetic outcomes, indicating high overall patient satisfaction. Both bone healing and prosthetic restoration showed positive results when surgical and prosthodontic cares were combined (Table 1 - see PDF).

## Discussion:

Mandibular fractures, which are frequently caused by falls, traffic accidents, or interpersonal violence, continue to be among the most common facial injuries seen in oral and maxillofacial trauma. Because these fractures affect essential functions like speech, mastication and appearance, they pose a serious clinical challenge. The variety of fracture patterns and related complications, in addition to their anatomical considerations, contribute to the complexity of mandibular fractures. Marker *et al.* assessed 348 cases of mandibular condyle fractures and stressed the significance of accurate diagnosis and customised treatment regimens to maximise results and avoid chronic dysfunction, including deviation during mouth opening or temporomandibular joint ankylosis [12]. A review by Boffano *et al.* highlighted the rising prevalence of pathological mandibular fractures, particularly in older patients whose bone quality is compromised by osteoporosis, infections, or neoplastic lesions, which further complicates the treatment strategy [13]. In their analysis of more than 2,100 mandibular fracture cases, Ellis and colleagues emphasised the prevalence of angle and parasymphysis fractures, which are often linked to the presence of third molars. This further supports the idea that impacted molars are a risk factor [14]. The fracture site, displacement, patient age and overall health all influence the management approaches for these fractures. In a military context, Thapliyal *et al.* described a clinical strategy that emphasises the prompt intervention and stabilisation of fractures with miniplates and intermaxillary fixation, especially in situations involving high-impact trauma [15]. It has been demonstrated that having mandibular third molars in the line of fracture increases the risk of fracture, especially in the angle region. Kandel *et al.*'s retrospective study supported this link and suggested that removing impacted third molars early on could prevent mandibular angle fractures [16].

Along with strict internal fixation techniques that enable early mobilisation and lower morbidity, modern management strategies also place a strong emphasis on early and accurate diagnosis. Particularly for displaced or comminuted fractures, Panesar and Susarla gave a thorough review of diagnostic techniques and available treatments, ranging from open reduction and internal fixation to conservative management [17]. Results have improved due to increased precision in hardware placement and fracture reduction brought about by advancements in imaging and surgical techniques. In order to successfully restore occlusion and function in patients with extensive mandibular damage, Bhandari *et al.* provided an example of full-mouth rehabilitation using implant-supported fixed prostheses after trauma [18]. Even with improvements in surgery, issues like infection, malunion, or non-union still pose a risk. The need for careful postoperative evaluation, particularly in patients with risk factors like weakened bone structure or excessive force application during surgery, was highlighted by Silva *et al.*'s report of a mandibular fracture after third molar removal [19]. In order to reduce complications and recurrence, systematic reviews such as the one conducted by Vian *et al.* emphasise the significance of choosing the best treatment modalities based on clinical evidence and patient-specific factors [20]. Wagner *et al.* also talked about pathological fractures that occur after third molar extractions and emphasised how crucial preoperative risk assessment and surgical planning are to preventing these kinds of incidents [21]. In conclusion, an interdisciplinary approach combining precise diagnostics customised surgical planning and patient-centered care is necessary for the successful management of mandibular fractures. In order to improve long-term functional outcomes and lessen the impact of maxillofacial trauma, new research supports the integration of preventative strategies like early rehabilitation and third molar management.

## Conclusion:

The effectiveness of a multidisciplinary approach in treating pathological mandibular fractures involving third molars is demonstrated by this study. High rates of bone healing, functional restoration and patient satisfaction were achieved through the combination of prosthodontic rehabilitation and surgical treatment. For such complicated cases, the efficacy of coordinated care is supported by stable results and few complications.
